# A Pain in the Neck — A Case of Intramedullary Spinal Ependymoma

**DOI:** 10.7759/cureus.7981

**Published:** 2020-05-05

**Authors:** Khushali Jhaveri, Manasa Veluru, Anusha Vakiti, Sandeep A Padala

**Affiliations:** 1 Internal Medicine, MedStar Washington Hospital Center, Washington, USA; 2 Hematology/Oncology, Medical College of Georgia, Augusta, USA; 3 Nephrology, Augusta University Medical Center, Medical College of Georgia, Augusta, USA

**Keywords:** spinal tumor, ependymoma, neck pain

## Abstract

Spinal cord tumors are sporadic and account for around 2%-4% of central nervous system neoplasms. Ependymoma is one of the most common spinal cord neoplasms and can present with different neurological signs and symptoms. They commonly present with neck or back pain and associated neurological involvement, with sensory symptoms usually antedating the motor symptoms. We now present a rare case of spinal ependymoma in a 26-year-old female who presented with isolated neck pain and the absence of other neurological symptoms. Attention to peculiar characteristics like isolated neck pain might be an essential key to early diagnosis and better prognosis.

## Introduction

Spinal cord tumors are exceptionally rare, accounting for around 2%-4% of central nervous system neoplasms [1]. Ependymomas are the most common amongst spinal cord neoplasms [2]. These tumors usually present with neck or back pain and associated neurological involvement, with sensory symptoms usually preceding the motor symptoms [3-4]. We now present a rare case of spinal ependymoma, which presented as isolated neck pain and no other neurological involvement [5].

## Case presentation

A previously healthy, 26-year-old female presented with an initial symptom of isolated neck pain. She denied any trauma, associated headache, nausea, vomiting, blurry vision, numbness, tingling, muscle weakness, or radiating pain. She did not notice any fever, fatigue, joint pain, changes in weight, or appetite. She did not recall any recent travel, sick contacts, or risk factors for human immunodeficiency virus (HIV).

On physical examination, the patient was afebrile, and her vital signs were within normal limits. On palpation of the neck, isolated neck tenderness was observed without any erythema, warmth, or swelling. Her active and passive range of motion was preserved. There was no nuchal rigidity, and Kernig and Brudzinki's signs were negative. Hoffman and Spurling's signs were negative as well. Her further neurological exam revealed 5/5 motor strength in all muscle groups in bilateral upper and lower extremities, and a sensory exam revealed intact sensation to light touch, pain, and temperature in all dermatomes. Vibration and proprioception were intact, and 2+ reflexes were noted in all muscle groups. Her chest, cardiovascular, abdominal, and extremities examination were unremarkable.

**Figure 1:**
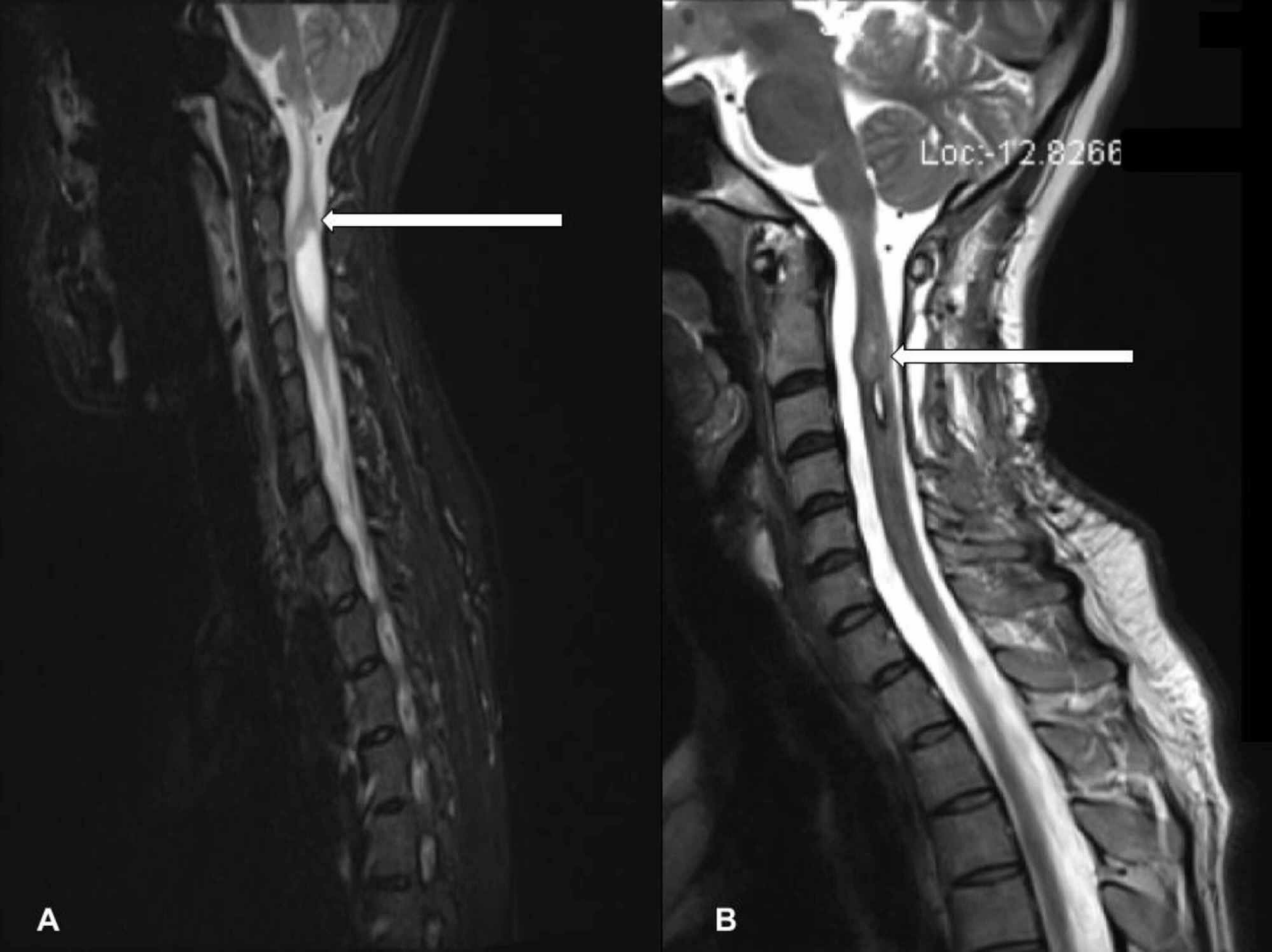
A. Heterogeneous intramedullary lesion with enhancing solid-cystic component from the mid-body of C2 to the upper body of C4 vertebral levels measuring 36 mm X 15 mm X 13 mm with the solid enhancing component of the lesion measuring 13 mm X 11 mm X 13 mm (CC X AP X Transverse). Perilesional edema and two tiny hemorrhagic foci, one at the cranial and one at the caudal aspect of the lesion. B: Residual tumor of 13 mm X 11 mm X 7 mm (CC X AP X Transverse) with the resolution of the cystic component. CC: craniocaudal; AP: anteroposterior

Initial laboratory examination, including complete blood count (CBC) with differential, inflammatory markers, and renal and liver function, were within normal limits. The X-ray of the cervical spine was done and was unremarkable. Given the benign neurological findings, a trial of muscle relaxants and anti-inflammatory medications was given. Due to the non-resolution of her symptoms for three weeks, MRI of the cervical spine was performed to rule out any cervical pathology, which revealed a heterogeneous intramedullary lesion at the mid-body of C2 to the upper body of C4 vertebral levels, raising suspicion for an intramedullary neoplastic lesion. Screening of the entire central nervous system (CNS) was otherwise unremarkable (Figure 1A).

To establish a specific diagnosis, the patient underwent a posterior cervical laminectomy. Gross total resection was not achieved due to a drop in sensory potentials during intraoperative neurological monitoring. Histopathological examination of the resected tissue showed glial neoplasm with hyalinized perivascular pseudorosettes with mild pleomorphism. No pseudopalisading necrosis or glomeruloid vascular proliferation was seen. These findings were consistent with World Health Organization (WHO) Grade 2 ependymoma.

The patient had no significant motor neurological impairment after surgery. However, a significant sensory and proprioceptive loss was observed due to a posterior spinal approach. She remains progression-free at two years with magnetic resonance imaging (MRI) showing a stable residual tumor (Figure 1B).

## Discussion

Neck pain is one of the leading causes of disability. With an annual prevalence rate of around 30%, it causes a significant burden to healthcare systems in the United States [6]. Most cases of acute neck pain resolve spontaneously; however, more than a third of patients continue to suffer from persistent pain and frequent recurrences [7]. While mostly benign, it can be an early presenting symptom for more grave illnesses. In such cases, a comprehensive history and physical examination remain at the cornerstone in identifying important 'red flags' that may assist us towards a severe pathology.

Intramedullary spinal cord tumors can exist with a diversity of symptoms. Neck or back pain is often the earliest symptom [3]. In patients with neurological involvement, sensory symptoms frequently antedate the motor symptoms and are consistent with the central location of the lesion within the spinal cord [4]. Our patient presented with an isolated symptom of neck pain without any other neurological symptoms [5]. As discussed, most cases of neck pain are benign and resolve spontaneously. In our patient, the non-resolution of symptoms, despite a trial of musculoskeletal relaxants, was concerning for a non-benign cause and led to further imaging. Her MRI revealed a heterogeneous intramedullary lesion, suspicious for an intramedullary neoplastic lesion.

It is essential to recognize and address such early symptoms judiciously, as further imaging can help not only in establishing a diagnosis but also in providing valuable details regarding tumor characteristics such as tumor size, presence or absence of cystic changes, and compression of vital structures. Such information regarding tumor characteristics is crucial as, along with presurgical functional status, it remains a significant predictor of postoperative prognosis [8-11].

Our patient underwent immediate surgery, which confirmed the diagnosis of WHO Grade 2 ependymoma. Due to the decrease in sensory potentials on intraoperative neuromonitoring, gross-total resection was not achieved. No significant motor neurological impairment was noticed after surgery. She sustained an expected decrease in sensory perception and proprioception in the setting of the posterior spinal surgical approach with marked improvement at one year from surgery [12]. Her residual tumor remains stable and without progression at a two-year interval on the most recent MRI.

## Conclusions

Cervical intramedullary ependymoma is a rare, slow-growing spinal cord tumor that can present with different, non-specific neurological symptoms. Neck pain, with usually benign differential diagnosis, can be an early presenting symptom for more grave illnesses such as ependymoma. It is necessary to identify peculiar symptoms like isolated neck pain, as it can be key to early diagnosis and better prognosis, as presurgical functional status is a significant predictor of postoperative prognosis.
